# A Review of Stimuli-Responsive Smart Materials for Wearable Technology in Healthcare: Retrospective, Perspective, and Prospective

**DOI:** 10.3390/molecules27175709

**Published:** 2022-09-05

**Authors:** Valentina Trovato, Silvia Sfameni, Giulia Rando, Giuseppe Rosace, Sebania Libertino, Ada Ferri, Maria Rosaria Plutino

**Affiliations:** 1 Department of Engineering and Applied Sciences, University of Bergamo, Viale Marconi 5, 24044 Dalmine, Italy; 2 Department of Engineering, University of Messina, Contrada di Dio, S. Agata, 98166 Messina, Italy; 3 Institute for the Study of Nanostructured Materials, ISMN–CNR, Palermo, c/o Department of ChiBioFarAm, University of Messina, Viale F. Stagno d’Alcontres 31, Vill. S. Agata, 98166 Messina, Italy; 4 Department of ChiBioFarAm, University of Messina, Viale F. Stagno d’Alcontres 31, Vill. S. Agata, 98166 Messina, Italy; 5 Institute of Microelectronics and MicrosystemsCNR–IMM, Ottava Strada 5, 95121 Catania, Italy; 6 Department of Applied Science and Technology, Politecnico Di Torino, Corso Duca Degli Abruzzi 24, 10129 Torino, Italy

**Keywords:** wearable sensors, pH sensors, sol–gel technique, health monitoring

## Abstract

In recent years thanks to the Internet of Things (IoT), the demand for the development of miniaturized and wearable sensors has skyrocketed. Among them, novel sensors for wearable medical devices are mostly needed. The aim of this review is to summarize the advancements in this field from current points of view, focusing on sensors embedded into textile fabrics. Indeed, they are portable, lightweight, and the best candidates for monitoring biometric parameters. The possibility of integrating chemical sensors into textiles has opened new markets in smart clothing. Many examples of these systems are represented by color-changing materials due to their capability of altering optical properties, including absorption, reflectance, and scattering, in response to different external stimuli (temperature, humidity, pH, or chemicals). With the goal of smart health monitoring, nanosized sol–gel precursors, bringing coupling agents into their chemical structure, were used to modify halochromic dyestuffs, both minimizing leaching from the treated surfaces and increasing photostability for the development of stimuli-responsive sensors. The literature about the sensing properties of functionalized halochromic azo dyestuffs applied to textile fabrics is reviewed to understand their potential for achieving remote monitoring of health parameters. Finally, challenges and future perspectives are discussed to envisage the developed strategies for the next generation of functionalized halochromic dyestuffs with biocompatible and real-time stimuli-responsive capabilities.

## 1. Introduction

The world’s aging populations are putting national healthcare systems under pressure due to rising hospitalization costs. On the other hand, the current pandemic has induced several countries to promote home-care programs that allow the elderly and patients with chronic conditions to be monitored remotely and are user and environmentally “friendly”, thus making periodic checks more pleasant and timely and providing clinical interventions only when their medical conditions require. For this purpose, in recent years, large investments have been made in both the research and industrial fields to provide innovative wearable devices and technologies able to monitor health conditions [1,2] and/or environmental parameters [3,4,5], resulting in the emergence of an important market segment. In fitness and wellness, the discrete and unobtrusive real-time monitoring of specific physiological, biomedical, and biomechanical parameters of athletes is of great interest in order to improve training compliance and/or competition performance [6]. In the same way, in the medical field, real-time and high-throughput devices for monitoring patients’ physiological parameters are even more desirable [7]. Unfortunately, the continuous monitoring of chronic medical conditions using implantable devices has significant drawbacks, such as the biodegradation of the sensing element and the patient’s immune system response to the host, e.g., changing diffusional barriers [8]. Wearable sensors can monitor physiological parameters in a non-invasive way, thus strongly reducing but not fully avoiding any reactions. They have attracted increasing interest and have been employed in many biomedical applications [9,10]. Moreover, they could potentially monitor both the wearer’s and the environmental parameters, thus acting as an intermediate between the body and surrounding conditions.

The main obstacle to the availability of the full technology, i.e., wearable systems and sensors for continuous health monitoring, is the lack of sensors [11,12]. The research is now focused on this issue since wearable body sensor networks (BSN) are an emerging medical sensor technology for achieving the unobtrusive monitoring of vital parameters in real time.

To minimize system failures when using such sensors, the sensing head must be in contact with the skin surface, and a fixing material (such as an elastic bandage or a tightly fitted undergarment) is used to keep it in place at the skin surface to avoid misplacement and background noise. Unfortunately, the use of a fixing material at the skin surface can cause measurement errors [13]. Another approach for in vivo and remote monitoring is the use of wireless micro-devices for signal transmission [14,15]. They are non-degradable and the electronics must be coated with biocompatible materials. Non-degradable micro-devices must be removed after the monitoring period and are not recommended for the post-operation control of physiological parameters, but rather are more indicated in chronic or long-term disease monitoring. Wearable electronics must be miniaturized, low-powered, and made of biocompatible materials in order to minimize the impact on the daily activities of the wearer. Through the integration of novel technologies, flexible support, such as textiles, can be equipped with information and power transmission capabilities and sensing functions as an infrastructure for embedded wearable microsystems [16,17,18].

Of paramount importance is the monitoring of the elderly and people with chronic conditions participating in “aging in place” programs. Wearable sensors have been used to monitor the recovery of patients after abdominal surgery [19]. The first step to achieving continuous monitoring in everyday life is correctly identifying the activities of daily living (ADL), i.e., walking, sitting, standing, etc. Many approaches have been followed mainly using accelerometers [20], for example, for step counting in patients with Parkinson’s disease [21]. An “in-shoe” pressure and acceleration sensor system [22] was developed to classify an arm movement coupled to the above-mentioned activities.

Several research projects have suggested that the continuous monitoring of ADL activities increases exercise compliance in populations at risk. As an example, the continuous monitoring of physical activities in obese individuals stimulates an active lifestyle, thus reducing clinical interventions [23,24].

Another use of the long-term monitoring of physiological data is for the diagnosis and treatment of cardiovascular diseases. Typical commercial technologies include the long-term monitoring of heart rate, oxygen saturation, blood pressure, body temperature, respiratory rate, and galvanic skin response, but some of these technologies are quite unpleasant. For this reason, clinical studies are currently validating wearable sensor platforms with the aim of improving the clinical management of patients with congestive heart failure [25]. For instance, the MIT Media Laboratory developed LiveNet, a system that measures 3D acceleration, electrocardiogram (ECG), electromyogram (EMG), and galvanic skin conductance for monitoring Parkinson’s symptoms and detecting epileptic seizures [26]. A custom data logger, “LifeGuard”, was developed to monitor the health status of individuals in extreme environments (space and terrestrial) [27].

The system has already been validated in hostile environments with good results. A wearable system to monitor brain activity using a non-invasive approach with fNIRS (functional near-infrared spectroscopy) has been recently developed [28,29] and patented [30] under the EU commission-funded project ASTONISH. The project AMON, again funded by the EU, developed a wrist-worn device capable of monitoring ECG, blood pressure, blood oxygen saturation, and skin temperature to monitor patients with cardio-respiratory problems [31]. Other projects worth mentioning that received grants from the European Commission are [32] MyHeart, WEALTHY, and MagIC. These projects were based on the idea of developing garments with wearable sensors for the health monitoring of people in home and community settings.

Wearable sensing systems can be broadly classified into two main categories: electronic devices fixed in various ways onto the fabric and chemical sensors fully integrated into the textile itself. The second category, certainly challenging but intriguing, allows for the production of a “smart” textile thanks to the full integration of the sensing system. The simplest approach is fabricating colorimetric sensors that cause a detectable color change in the cloth. This is achieved using halochromic dyes and they have been used for employees working with chemicals; the color change immediately alerts the workers of a potentially harmful chemical leak without requiring power sources [33,34,35,36]. Their main drawbacks are the relatively low stability and pH sensitivity, which are quite important at high gas concentrations. To overcome the last limit, most textile sensors have been fabricated with a large surface area, i.e., through electrospinning, thus improving the pH response [37,38,39]. For example, Agarwal et al. [40] developed universal pH sensing nanofibrous sensors using various halochromic dyes with Nylon 6; Pakolpakçıl et al. [41] used natural halochromic dye with sodium alginate and a polyvinyl alcohol mixture to formulate pH-indicating nanofibrous sensors; Guinovart et al. fabricated an electropolymerized polyaniline (PANi)-conducting polymer for the production of a bandage-based wearable potentiometric sensor for monitoring wounds’ pH [42]; Kassal et al. developed a wireless RFID-based smart bandage for the optical determination of pH by embedding covalently modified cellulose particles with a pH indicator dye in a biocompatible hydrogel [43]; Getmeyer et al. [44] detected gaseous NH_3_ and HCl with nanofibrous sensors fabricated using electrospinning and sol–gel methods; and Jeevarathinam et al. [45] and Suleymanov et al. [46] used an aggregation-induced emission of dyestuff.

To commercialize smart textiles, the main areas that need to be addressed are service life, washability, productivity, and production costs while maintaining a high detection performance with respect to liquid or gaseous alkalis and acids.

The purpose of this retrospective, perspective, and prospective review is to provide an overview of the advancements in the field of wearable sensors with a focus on colorimetric sensors embedded into textiles. In particular, a thorough discussion on flexible electronics and different wearable sensor categories and applications is dealt with in detail in this review.

## 2. Flexible Electronics

Over the past ten years, the Internet of Things (IoT) has seen exponential growth as markets recognize the true potential of real-time data acquisition for a range of applications in entertainment, knowledge dissemination, defense, the environment, and healthcare [47,48]. Since the real-time monitoring of physiologically relevant indicators is essential not only in urgent hospital settings but also during regular daily activities, the medical applications of the IoT have attracted the most attention [49]. Such ongoing essential information can notify the user of health problems so they can take preventative measures and avoid life-threatening medical conditions.

Wearable sensors could become a key component of the IoT in healthcare since they offer new ways to monitor people continuously and give the wearer individualized access to crucial physiological information about their health [50]. Due to the advancement of flexible electronics, wearable sensors are no longer just confined to on-body applications but may also be integrated with other surfaces, such as those of buildings or vehicles, for far wider applications. Flexible electronics have been theoretically possible for many years. In theory, anything long or thin can stretch to become flexible. Although flexible cables and wiring are the best examples, it was not until the space race that silicon wafers used in satellite solar cells were thinned to increase their power-to-weight ratio, allowing for some warping. This concept permitted the first flexible solar cells in the 1960s [51]. The development of conductive polymers [52], organic semiconductors, and amorphous silicon [51,53] led to significant advancements in flexibility and processability over the ensuing decades, and as a result, they were used as a base for electronic devices in applications that required properties such as bending, rolling, folding, and stretching that could not be met by conventional electronics [54].

New materials and fabrication methods that enable the direct production of high-performance, scalable electronic devices on flexible substrates are currently of significant interest. This interest now includes qualities such as stretchability and the capacity to heal, which can be attained by using elastomeric substrates with strong molecular connections. [55,56]. In a similar vein, biocompatibility and biodegradability have been made possible by polymers that have no negative effects on the body and can degrade into smaller constituent parts after use [57,58,59]. Devices that can conform to dynamic, complex surfaces, such as those found in biological systems and soft robotics that are inspired by nature, are now possible thanks to recent advancements.

New applications for these next-generation flexible electronics include flexible lighting and display technologies for consumer electronics, architecture, and textiles; wearables with sensors that track people’s habits and health; implantable electronics for better medical imaging and diagnostics; and enhancing the functionality of robots and unmanned aircraft with light-weight and conformable energy-harvesting devices and sensor technologies.

Depending on the context, flexibility could mean several things. From the folding, twisting, stretching, and deforming needed for devices in electronic skin to bending and rolling, enabling better handling of large-area photovoltaics while maintaining device performance and dependability. Although there has been some early progress and significant discoveries, the field of flexible electronics still faces numerous obstacles before becoming a part of everyday life. This offers a tremendous opportunity for scientific research and development to gain significantly and quickly more insights and progresses into the field of wearable sensors.

Despite the advantages of wearable sensors, research and development in this area have advanced unevenly. Early research efforts concentrated on developing wearable sensors that can measure temperature, body motion, or ECG. Current wearable devices frequently track the user’s physical activities and vital indicators (such as heart rate) [41,60]. However, in order to fully understand a wearer’s health, performance, or stress at the molecular level, continuous chemical parameter monitoring is essential. In light of the importance of wearable sensors, various groups have recently discussed significant advancements in this area of research [61].

Thus, devices that can potentially monitor wound healing [62], electrolytes [63], metabolites [64], heavy metals [65], and toxic gases [66] directly on the body in various biofluids, such as sweat [67], tears [68], and saliva [69] have been demonstrated.

Electronic textiles cover a wide range of textile items that can incorporate electronics, including filaments and fibers. They differ from conventional electronic approaches in that they are physically flexible and have a characteristic size [70]. Electronic fabrics’ unique qualities make them easily adaptable to the sensing needs of wearable chemosensors. Scientists have created a variety of methods and materials for the design and production of smart textiles with a range of features and functions over the past ten years. These electronic textiles are constructed using a variety of different processes, including weaving, sewing, and embroidery.

Wang et al. [71] have developed a wearable, flexible, and stretchable glove-based electrochemical biosensor for the detection of organophosphorus chemicals, presenting the first design for performing fingertip enzymatic assays. The sampling and biosensing processes are carried out by the glove-based sensor using different fingers; the enzyme is fixed to the index finger and the thumb is used to collect residue. An enzyme-immobilized biosensing detection finger, a sampling finger, and wireless real-time data transmission to a smartphone are all included in this adaptable, wearable “lab-on-a-glove”.

Chemical coatings for creating electronic fabrics have recently been developed (e-textiles) [72] and have undergone intensive research to create the next generation of wearable electronics. These electronics have exciting potential applications in portable military equipment, medical monitoring gadgets, and smart fabrics with built-in electronics. In order to achieve this, the deposition of carbon nanotubes (CNTs) onto textile fabrics has been researched. CNTs are ideal for smart textiles due to their exceptional aspect ratio, incredible structural flexibility, high mechanical qualities, and excellent thermal and electrical conductivity [73]. Using photoplethysmography (PPG), a popular method for gathering key physiological data, such as heart rate, respiration cycles, and blood oxygen saturation, the potential use of CNT-treated cotton fabrics as conductive materials for signal transmission has been investigated [74].

## 3. Wearable Sensors

Physiological measurements of interest in rehabilitation and health monitoring include blood pressure, blood oxygen saturation, heart and respiratory rates, and muscle activity. The aim is to provide an indication of health status that can also be useful to formulate a diagnosis. The growth of the wearable technology field allows for performing continuous monitoring of physiological parameters at home, whereas before, this was possible only in a hospital setting.

**Figure 1:**
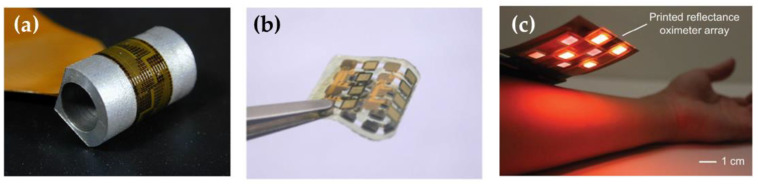
(**a**) Silicon flexible skin wrapped around a half-inch diameter aluminum block, (**b**) folded silicon skin (a and b adapted from [23]). (**c**) flexible oximeter composed of arrays of OLEDs and OPDs to measure oxygenation of blood and tissue (adapted from [24]).

The main issue to address is the sensor stability and response accuracy over time. Chemical sensors need to be in contact with bio-fluids to operate, thus exposing the device to biofouling, oxidation, or chemical changes. In this perspective, optical and electrical sensors are considered more robust, especially if they are fabricated using proven materials. The sensing device must be comfortable for the user, hence many approaches to producing robust and “comfortable” devices have been proposed. As an example, a “silicon flexible skin” obtained using silicon islands integrated with boron-doped strain gauges and metal pads was proposed by Katragadda et al. [75]. This approach would allow exploiting the mature silicon technologies fully. In Figure 1a, a flexible silicon skin wrapped around a half-inch diameter aluminum block is shown.

The natural evolution of such an approach brought by flexible electronics has experienced tremendous growth thanks to the recent advances in printable materials and techniques [76]. In fact, silicon ICs, which can be used for real-time data processing, wireless communication, and display visualization can be integrated into platforms. Thanks to these new products that are more easily integrated into textiles and with the progressive miniaturization of Si-based devices, large-scale setups designed for clinical or laboratory use are becoming portable. The integration of optical sensing systems with consumer electronic goods is already a fact; a good example is wrist-mounted wearables. They can measure heart rate, heart rate variability, and blood oxygenation [77]. Among the physiological parameters, heart rate is one of the most often measured, with healthy individuals having a heart rate of 60–100 beats per min (at ≈21 mmHg pulse pressure) [78]. Heart rate can be measured by PPG-based technologies [79,80], sound-based techniques [81], etc.

Wearable PPG sensors are commercially available (e.g., Apple Watch, Fitbit, and Samsung Gear) and instantaneously monitor heart rate. They are mainly formed by a light-emitting diode (that can be easily miniaturized [82]) and a photodetector. The physical principle is based on the optical properties of the tissues and blood. Most of our biological tissues transmit, reflect, and scatter, according to the Beer–Lambert law, visible and near-infrared (NIR) light [83].

One of the main drawbacks of these devices is signal distortion due to motion artifacts, which can be more challenging if application during physical activity is envisioned. To overcome this problem, short wavelengths (400–600 nm) are quite often selected for LEDs used for PPG measurements. Such light does not penetrate the tissue deeply, hence does not reveal much cardiac activity and blood vessel information, but the measurements are less affected by artifacts due to movement thanks to the short light path.

**Figure 2:**
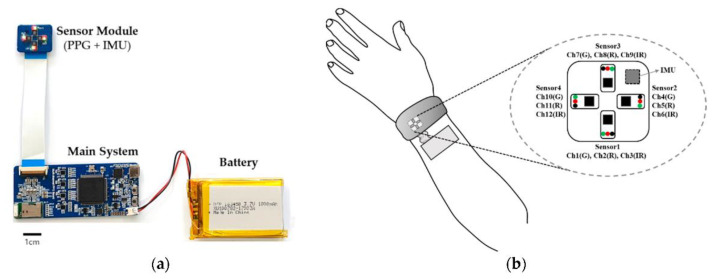
Wearable PPG sensors: system consisting of a main system, sensor module (PPG and IMU), and battery (**a**); schematic of direction and wavelength (G: green; R: red; IR: infrared) of the sensor for each channel when the system is worn on the wrist (**b**). (adapted from ref. [84]).

On the other hand, information on blood vessel status and heart activity can be obtained only using long wavelengths since their penetration in the tissues is deeper. Recently, Lee and coworkers [84] showed a strong reduction of motion artifacts by combining multiple LED wavelengths (visible and IR) coupled with an algorithm for signal processing (Figure 2).

By implementing a more complex system, functional near-infrared spectroscopy (fNIRS) can be made wearable. The physical principle behind this technique is the same as PPG. It is known that hemoglobin in its two states, oxygenated (O_2_Hb) and deoxygenated (HHb), is the main chromophore absorbing light in the NIR spectral range with different spectra [85]. These characteristics allow the monitoring of the backscattered NIR light to determine the blood oxygenation value [86].

By performing a continuous analysis, the back-scattered photons provide information related to the dynamical changes of O_2_Hb and HHb concentrations in the blood (both hemodynamic and metabolic). If applied to the brain, fNIRS can provide insights into neural activity [87]. Measurements are performed by irradiating the scalp at two different wavelengths in the range of 700–950 nm (one above and one below 800 nm) and collecting the light in a photomultiplier tube. Optical fibers are used both to irradiate the scalp surface and collect the back-scattered light [88].

**Figure 3:**
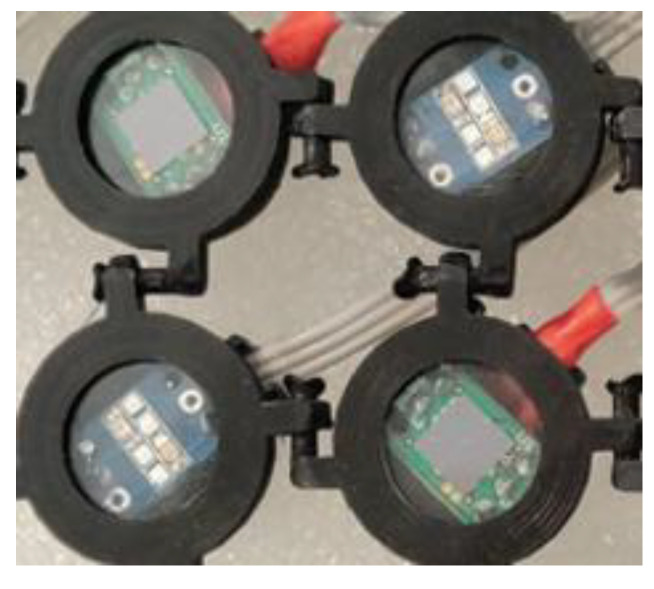
Details of SiPMs (top left and bottom right) and LEDs (top right and bottom left) for diffused optical tomography/functional near-infrared spectroscopy (DOT/fNIRS).

Silicon photomultipliers (SiPM) [89,90,91], which are proposed as optical detectors in many sensing systems [92,93,94,95], and miniaturized LEDs are combined in a potentially portable system [96,97,98,99] operating in continuous waves. For each cycle, each detector collects the signal coming from the LEDs placed on the scalp (each LED is switched to a frequency of 20 Hz). The system performs measurements of the O_2_Hb and HHb concentrations in the brain region defined by the LED-SiPM position, and the relative distances between the emitter and the receiver regulate the penetration depth. In a recent patent [30], the same authors proposed the use of an optimized circuit and algorithm for optimal system operation to be used in diffused optical tomography (Figure 3).

**Figure 4:**
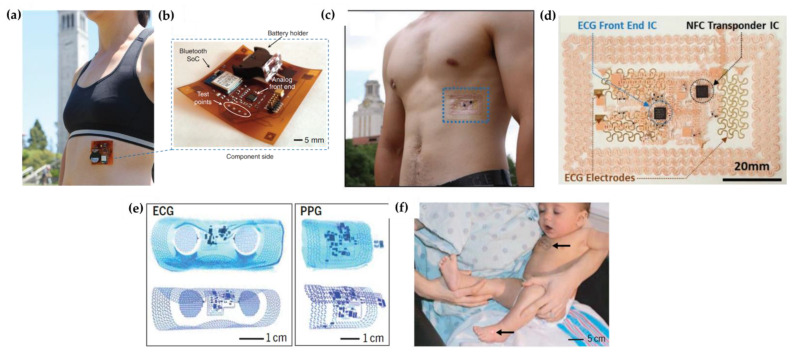
Wearable ECG sensor systems. (**a**,**b**) ECG electrode pair and a printed thermistor. (**a**,**b**) Reproduced with permission. [103] Copyright 2016, Wiley-VCH. (**c**,**d**) A peel-and-stick form-factor wearable ECG patch. (**c**,**d**) Reproduced with permission. [104] Copyright 2019, Wiley-VCH. (**e**,**f**) Wearable ECG and PPG sensors for neonates. The arrows show sensor placement locations. (**e**,**f**) Adapted with permission. [105] Copyright 2019, AAAS.

Finally, many studies on wearable ElectroCardiogram (ECG) sensors can also be found in the literature [100,101,102]. Through ECG, both the rate and regularity of heartbeats can be monitored and the system can be made wearable. As an example, ECG signals were measured by Khan et al. [103] (with gold electrodes) and Jeong et al. [104] (Figure 4a–d, respectively) using printed sensors. In the first system, a Si-based integrated circuit processes the sensor data and transmits it via Bluetooth to the cloud. In the second system, a wireless circuit was also integrated to transmit the sensor data. Finally, Chung et al. [105] combined both ECG and PPG for the simultaneous monitoring of cardiac parameters in neonates (Figure 4e,f). Moreover, in this case, the data are transmitted wirelessly to the cloud.

Of great interest are biochemical sensors to monitor both subject biochemical signals and chemical compounds in the environment. Biochemical sensors require the device to come in contact with body fluids to be analyzed and may require the collection and disposal of such fluids. Hence, they may have a complex design but great improvements in micro- and nano-fabrication technologies [106] have been a strong driving force for their diffusion. As an example, a minimally invasive wearable closed-loop quasi-continuous drug infusion system was designed by Dudde et al. [107] to measure blood glucose levels, which is achieved using a novel silicon sensor that measures glucose levels in real time using a microperfusion technique. The system is also composed of an integrated Bluetooth communicator to connect it to the cloud. It is a two-way communication system that can upload the logged data and receive instructions from a personal digital assistant (PDA).

Biochemical sensors can also be coupled in an array, as demonstrated with the BIOTEX project supported by the European Commission. The aim of the project was the integration into textiles of biochemical sensors to monitor body fluids. A textile-based system was developed to collect body sweat, coupled to a sensor array to perform the testing of pH, sodium, and conductivity either in vitro or in vivo [108]. The researchers demonstrated that the system could be used for real-time sweat analysis during physical activity. The project ProeTEX [109] aimed to develop a wearable sensitive garment for firefighters, integrating CO_2_ and temperature sensors to test environmental conditions with a set of sensors to monitor the operator for movement, body temperature, position, blood oxygen saturation, heart rate, and respiration rate. The system warns the firefighter of a potentially dangerous environment and, at the same time, provides information about their health status to the control center. The systems developed in the above-mentioned projects rely upon the design of robust e-textile-based wearable systems for remote health monitoring applications. These projects prove the growing interest in the development of self-contained lab-on-a-chip systems. These systems are an example of how point-of-care medical testing and diagnosis can be revolutionized by making testing and diagnosis easy for the patient, thus increasing their quality of life. Other examples are provided by Wang et al. [110] who developed a system-on-chip (SOC), integrating pH and temperature sensors for remote monitoring applications, including a frequency-shift keying RF transmitter, a sensor interface, an ADC, a microcontroller, and a data encoder; and by Ahn et al. [111], who developed a plastic lab-on-a-chip device for the biochemical detection of blood gas concentration and glucose, which was low-cost and disposable. For blood sampling, the device uses a passive microfluidic manipulation system, which is less complex than active microfluidic pumps.

There has recently been a gradual trend toward using “smart materials” to achieve desirable material characteristics. A smart material is a new version of the material with improved conventional functionality. In addition, due to their natural intelligence properties, smart materials have adaptive capabilities to respond to various stimuli and environmental changes. In other words, smart materials have the capability to alter their physical characteristics as a response to specific stimulus factors [112].

### 3.1. Chromogenic Materials

**Figure 5:**
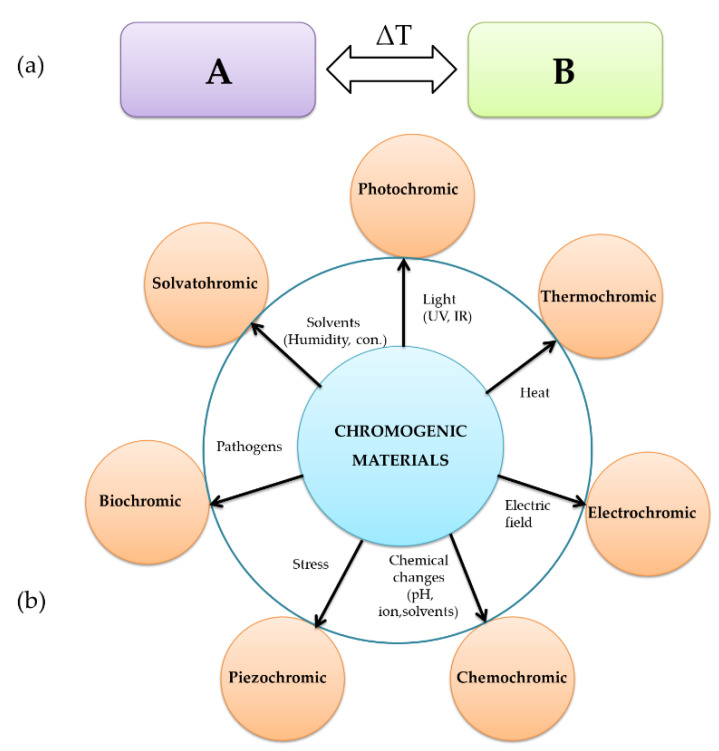
Color change of chromogenic materials from various stimuli (**a**) and examples on the basis of the stimuli involved (**b**).

Our daily sensorial experience is mainly based on sight and colors and their changes are easily understood. Some natural or synthetic materials exhibit optical changes (color, transparency, reflectance) [113] through induced stimuli (temperature, humidity, brittleness, pH, ionic properties, chemicals, etc.) [114] (Figure 5a). These compounds do not change their color; instead, they change their optical properties and thus they are described as color-changing materials and referred to as “chameleon” or “chromogenic materials”.

Chemically, the chromogenic property is the result of a phenomenon called chromism, in which a reversible color-changing phenomenon usually occurs in a material matrix through a change in the molecular density, particularly in a p- or d-electron state [115]. Capable of responding to various stimuli, chromogenic materials are a crucial component of smart materials. They are the active element of sensors/indicators capable of detecting and distinguishing changes in the environment or a system of interest (such as packaging, skin, biosubstrates, etc.).

Color changes can be used in color-changing sensors, allowing a chemical analysis (at least qualitative) to be conducted by the naked eye [116]. Based on the stimulus origin, the color-changing process in smart materials is mostly classified into photochromism, electrochromism, thermochromism, mechanochromism, solvatochromism, chemochromism, and biochromism [117] (Figure 5). 

The use of chromogenic materials has been widely considered in various fields. As shown in the review of Sadeghi et al. [118], chromogenic materials have become an area of interest in a broad range of academic fields. Among the peer-reviewed articles listed in Elsevier’s Scopus database, using the keywords “chromogenic materials”, manuscripts concerning the medical field show the highest number of records (21.4%). The application of chromogenic polymers is an emerging approach for smart health monitoring. Chromogenic surfaces can be considered a clever communication method that alerts doctors upon warning signs in the patient’s health.

### 3.2. Photochromic Materials

Photochromic materials change their color reversibly when exposed to light, going from colorless to colorful or switching between two distinct colors. Given that sunlight’s ultraviolet (UV) and infrared (IR) radiation can significantly alter the optical characteristics of photochromic materials, consideration should be given to the surroundings. Afterward, when the stimulus (light source) is removed, these materials irreversibly return to their original color [119]. 

The color-changing phenomena typically happen when radiation energy causes a change from a thermodynamically stable state A to a metastable state B. When the heat or radiation source is turned off, state B then reversibly transforms back into state A. By absorbing light’s radiative energy, colorless or partly colored photochromic materials reorganize the bonds between their atoms [120].

Despite a wide range of synthesized organic and inorganic photochromic materials, only a small number have been commercialized, with organic photochromic materials being the most common. Accordingly, spiro-pyrans, spiro-oxazines, and naphtho-pyrans are commonly used as commercial organic photochromic dyestuffs [121]. 

The photochromic properties of these materials are strongly related to the aromatic rings, as well as to the functional groups bonded to the rings, which might introduce an extensive color gamut. For example, upon UV light irradiation, the bond between spiro-carbon and oxazine is broken and the carbon-oxygen ring is opened [122]. After the UV light stimulus is withdrawn, photochromic materials gradually change back to their original structure by releasing the absorbed energy (heat and/or light), finally forming the ring again. High temperatures can accelerate material decomposition and cause strong color changes but the photochromic response can be weakened [123].

Some inorganic compounds also exhibit chromogenic properties, including copper, mercury, metal oxides, and various halides such as silver, zinc, and yttrium halides. These materials coat metal, glass, and ceramic surfaces effectively and are more durable than inorganic materials [124].

Their main drawback is their durability since they are insufficiently stable when exposed to long-term light stimuli in outdoor applications. Moreover, high-speed color switching and precise color classification need to be further investigated. To utilize photochromic materials as chromogenic compounds, they should be appropriately incorporated into a wide range of materials such as polymer resins, inks, and paints [125].

### 3.3. Thermochromic Materials

Thermochromism is commonly based on the chemical equilibrium between two different forms of a molecule or between different crystalline phases [126]. A successful example of a thermochromic material product on the market is the temperature indicator used in many everyday products, for example, mugs (logo or words on the mug); color-changing plastics; ceramics; spoons for children; indicator stripes on beer, beverage cans or bottles; milk cartons; umbrellas; golf balls; jewelry; cosmetics; and even toilet seats [127].

Thermochromic materials reversibly (or irreversibly) change their color, color intensity, or transparency in response to a variation in temperature [128]. The thermochromic phenomenon is displayed either gradually based on a slow temperature increase (continuous) or abruptly at a specific temperature (discontinuous). This phenomenon is called thermochromic switching or transition temperature, which can be modified based on doping and straining.

Thermochromic materials can present themselves as [129,130]:

1. organic materials.
2. inorganic and metallic compounds.
3. polymeric materials.
4. thin films with transition-metal oxides.

In the first case, thermochromism in organic compounds is related to the variation of the state of equilibrium in the molecular species. Thermochromic organic materials can be categorized into three major types. Some liquid crystals transition from a crystalline solid equilibrium state to an isotropic liquid state in each temperature range. Thanks to distinct and accurate color changes caused by temperature variations, thermochromic organic compounds are widely used in different industries for different purposes such as photo-storage instruments, optical sciences, optical sensors, and textiles. There are some drawbacks related to the application of organic compounds as versatile thermochromic materials, including their low color density, low molecular weight, and occasionally high melting point for the change in equilibrium (color-changing capability) [131].

As far as inorganic and metal compounds are concerned, owing to the presence of several mechanisms, including the change of the ligand geometry or the type of solvents, certain metallic and inorganic compounds exhibit a color-changing process in the solid or liquid state. However, these materials mostly exhibit thermochromic phenomena in solution at high temperatures, which limits their practical applications.

Regarding polymeric materials with naturally thermochromic properties, they are usually classified into two groups. The first includes polymers having helical superstructures, i.e., liquid crystalline or conjugated polymers, whereas the second consists of polymers with thermochromic agents (inorganic dye/pigments and conjugated polymers) incorporated or resulting from the integration of thermochromic and polymer compounds [132].

Finally, sol–gels with thermochromic transition-metal oxides, such as Ti_2_O_3_, VO_2_, V_2_O_3_, V_2_O_5_, Fe_3_O_4_, and Mo_9_O_26_, can also be described as thermochromic materials owing to their reversible transition phase ability and variation of their crystalline structure based on temperature. The transition temperature can be adjusted through doping with the appropriate metal ions. To reduce the transition temperature in metals with high transition temperatures to extend their applicability, doping with high-valent transition metal ions, tungsten, niobium, or titanium ions can be implemented. As an example, VO_2_ exhibits a significant reduction in the transition temperature from 70 °C to 25 °C through the incorporation of a 2% tungsten ion. In contrast, some cations, such as Cr^3+^ and Al^3+^, with larger atomic radii than V^4+^ ions can lead to an increase in the transition temperature. Such materials are desirable in industry and academia because they can produce different color states at different temperatures. As such, they can be widely used in industries owing to their ability to repeatedly change from their original color to a new colored state [133].

### 3.4. Electrochromic Materials

Electro-active compounds with changing, evocating, and bleaching colors using appropriate electrochemical stimuli, such as the redox process (electron transfer), are called electrochromic materials [134] (Table 1). Oxidation-reduction reactions during transmittance and/or reflectance are the main mechanisms for the color-changing phenomenon in these materials. 

The production of various visible regions for electron absorption bonds allowing the switching between redox states leads to electro-chromism [135]. Most of the color-changing processes occur either between a colored state and a colorless (bleached and transparent) one or between two colored states, whereas polyelectrochromic compounds have more redox states, thus providing changes among several colors (Table 1). 

Inorganic electrochromic materials are generally classified into three types [136]:

1. cathodically coloring materials that are colorless in the oxidized state and colored in the reduced state such as WO_3_, MoO_3_, and TiO_2_.
2. anodically coloring materials that exhibit color in the oxidized state and are colorless in the reduced state such as NiO, PB, and IrO_2_.
3. coloring materials that are colored in both oxidized and reduced states such as V_2_O_5_, CoO_x_, and Rh_2_O_3_. Also, polythiophene, polypyrrole, and pyrazoline (conjugated and electro-active polymers) can exhibit electrochromic processes.

Accordingly, their applications in research and industry will increase not only because of their high color tenability and optical contrast but also because of their convenience in the synthesizing process and their thin-film-forming properties [137].

Upon exposure to sufficient electro stimuli, electrochromic-conjugated polymers change the k_max_ and π–π transitions, thus turning from a colorless neutral state (transparent) into a colored charged state. The color-changing process in a conjugated polymer occurs because of the nature of the energy gap in the side or main chain of the repeating unit. In the following section, the preparation procedures of a few important electrochromic materials are highlighted. 

Polyaniline exhibits four different colors based on the different oxidation states: black for pernigraniline, yellow for leucoemeraldine, blue for emeraldine base, and green for emeraldine salt [138]. It is prepared using chemical or electrochemical oxidation. In the latter approach, the electrode is immersed in hydrochloric acid with a low concentration of aniline, thus obtaining a thin film on the electrode. 

Polypyrrole, which is naturally bright-yellow-colored and becomes darker after air oxidation, is prepared through pyrrole polymerization. If doped, its natural color is blue or black based on the thickness and degree of polymerization. Polythiophenes containing sulfur heterocycle show promising conducting properties after oxidation [139], and their optical properties are related to the diversity in the counter-ions and functional groups bonded to the sulfur heterocycle. 

They show a significant color shift in response to temperature changes, solvent properties, and binding with various molecules. Pyrazolines containing five heterocyclic groups and two nitrogen atoms have a functional ring unit with an electron-accepting imine (–C=N–) group and an electron-donating (–N(Ar_1_)–) group. Their physicochemical and optical characteristics are strongly dependent on the nitrogen groups, which can accelerate the electron transfer, thus resulting in nonlinear optical, ferromagnetic, and conducting properties in pyrazolines [140]. 

Currently, pyrazoline and its derivatives are being considered in a wide range of fields such as OLEDs, nonlinear optical devices, solar cells, and fluorescence brighteners [141]. Electrochromic materials can be successfully applied to various products owing to their color-changing and electrical conducting abilities.

There is a broad range of chemicals with electrochromic properties being investigated for potential use in commercial products.

### 3.5. Ionochromic Materials

Materials with a color-changing ability (from colorless to colored or from colored to colorless) by inducing ionic species in an ionic state are indicated as “ionochromic” compounds and if they exhibit this behavior, they are called ionophores. The ionochromic process occurs through a reaction between the ionochromic compounds and negatively charged anions (Table 2).

These materials exhibit a reversible color change from the original color to a new one. When the stimulus is withdrawn (i.e., the ion inducing the ionochromic response is removed), the compound returns to its original color [142,143]. The ionochromic process can occur based on different mechanisms, including the following [137]:

1. halochromism owing to a change in acidic or alkaline pH;
2. acidochromism stimulated by acid;
3. metallochromism deriving from the formation of colored complexes from metal ions and chelating ligands.

There is a wide range of organic molecules with ionochromic properties, including commercial dyes and pH indicators, such as phthalides, leucotriarylmethanes, and fluorans. 

Moreover, acidic protonation color-changing materials such as azo dyes and styryl dyes, merocyanines and indophenols, metal ions, and particularly transition-metal ions, can result in the creation of metallochromism complexes through a reaction with the chelating ligands, thereby altering the color.

Many chemical structures in pH-sensitive dyestuffs can react with a wide range of pH levels and such materials can thus be used as an optically changing chemical in titrations. pH-sensitive dyes have been commonly applied as reversible indicators in acidic or basic conditions through an ionic-responsive process. 

**Figure 6:**
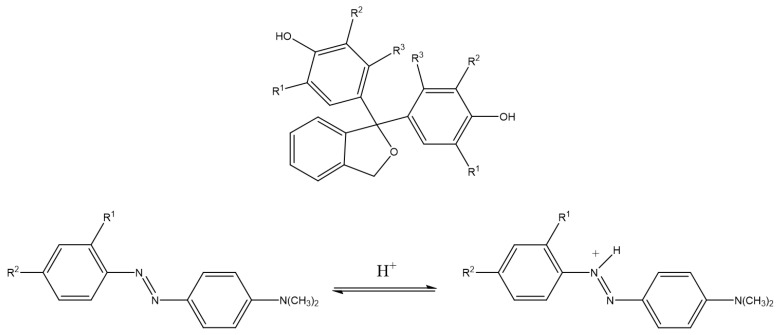
The functional groups’ positions in the phthaleins and sulfophthaleins azo dyes.

Various phthaleins and sulfophtaleins with different chemical functional groups (Figure 6) and azo dyes with their unique pH ranges, can be used to produce analytical indicators and sensors. 

Although pH-sensitive dyes have been commonly prepared as commercial products (Table 3) because of their carcinogenic potential, some azo dyes, such as Methyl Yellow and Congo Red, are no longer used [144].

Dyes sensitive to a wide range of pH can be used as optical change agents in different physical environments including as moisture indicators in the presence of a pH modifier such as carboxylic acid (used in hydrochromic ink).

These materials are often used in species (ionic, acidic, or donor) and are induced by light or heat stimuli.

### 3.6. Mechanochromic Materials

Mechanical stresses represent an appropriate stimulus by interacting with chemical compounds in different forms. Considerable efforts have been directed at investigating the nature of the interactions between molecules and mechanical stimuli.

For this purpose, different methods have been developed at different scales ranging from supermolecular structures to single molecules. The compounds and systems that display optical-changing properties when subjected to mechanical stimuli are called mechanochromic.

**Figure 7:**
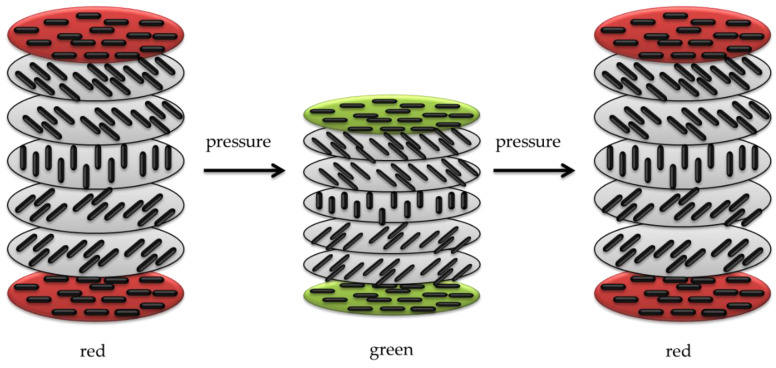
Schematic of mechanochromisms.

The mechanical color-changing phenomenon is mainly considered a change in the transmitted and emitted wavelengths (Figure 7). However, this process is occasionally based on the emission intensity rather than the wavelength and fluorescence lifetimes.

When mechanochromism is based on transmission and emission wavelengths, it requires a light source in the ultraviolet or visible range [145]. Mechanochromic compounds have been widely used as stress sensors, e.g., in situ failure indicators for fractures, corrosion, fatigue, or creep [146]. Materials with mechanochromic ability under physical stimuli encompass a wide range of materials including polymeric compounds or inorganic materials. Various stresses, such as milling, friction, crushing, rubbing in the solid state, and high pressure or sonification in the solid or liquid state, can be regarded as potential mechanical stimuli. Mechanochromism can be classified into two categories based on the mechanical stimuli and color-changing mechanisms [147]:

1. Piezochromism, based on pressure stimuli. Piezochromic compounds are mostly prepared from conjugated polymeric materials. The color-changing property in the solid state is typical of three classes of compounds, namely, polydiacetylenes, polythiophenes, and polysilanes. Pressure and compression cause perturbations of the ground and excited states, changes in the crystal structure through first-order phase transitions, or changes in the molecular geometry in species that comprise the solid [148].
2. Tribochromism, induced by friction or grinding. Tribochromic materials include spiropyran, spirooxazine, and thioindigo, and exhibit strong colored states. Besides organic compounds, some inorganic materials show mechanical color-changing properties including palladium complexes and single crystals of NaCl, CuMoO_4_, and LiF. However, inorganics require exposure to high pressure, in the order of thousands of MPa, to show a reaction and this is a major obstacle to their application as pressure-responsive compounds. In fact, a relatively low pressure-responsive ability is usually considered an economic factor in facilitating the application of reversible mechanochromic compounds. Occasionally, the modification of the chemical composition can enhance their performance such as substituting a small portion of Mo with W in CuMoO_4_. This causes the mechanical responsive capability of CuMoO_4_ to shift from 250 to 20 MPa [149].

Several studies and patents have been reported on mechanochromic materials, including security inks [150], stress and crack sensors or indicators [151], biological [152] or healthcare applications [153], and cosmetics. Thermochromic inks can also respond to mechanical stresses such as rubbing with the fingers, which causes the ink to disappear or change visually. Seki et al. developed a reversible mechanochromic compound by modifying aryl gold isocyanide complexes. In this process, a 9-anthryl gold(I) isocyanide complex showed a bathochromic shift from blue (448 nm) to the IR region of 900 nm when mechanically [154] ground. This compound can be utilized in bio-imaging and security ink because it can emit infrared light, which is invisible but detectable using a spectrometer.

### 3.7. Solvatochromic Materials

**Figure 8:**
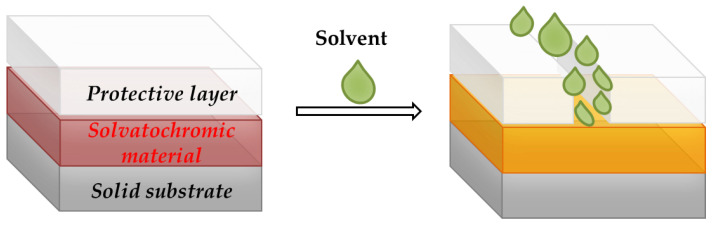
Schematic representation of a solvatochromic sensor system.

Certain materials display different colors depending on the solvent they are dissolved into. This mechanism is called solvatochromism. Materials with various chromophore groups show solvatochromic behavior by changing the visual adsorption and emission spectra (optical properties) due to the interactions between the solvatochromic material and solvent [137] (Figure 8).

Due to different solvent polarities, the ground and excited states of the solute chromophore are modified, leading to changes in the energy gaps (dipole moment) between the two electronic states. The color-changing performance in a solvatochromic system is due to variations in the position, intensity, and shape of the absorption spectra through the interaction between the solvatochromic material and the solvent. Currently, the same concept of “solvent-based effect” is also exploited for gels, polymers, micelles, films, and various surfaces [155]. Depending on the solvent polarity, positive and negative solvatochromism can be detected. It can be classified into two types based on the variations in the dipole moment between the ground and excited states of the chromophore [156]:

1. Negative solvatochromism with increasing solvent polarity is regarded as a hypsochromic (or blue) shift, e.g., 4-(40-hydroxystyryl)-*N*-methylpyridinium iodide appears red, orange, or yellow in 1-propanol, methanol, and water, respectively.
2. Positive solvatochromism with increasing solvent polarity is regarded as a bathochromic (or red) shift, e.g., 4,4′-bis(dimethylamino)fuchsone appears orange in toluene, red in acetone, and red violet in methanol.

Owing to the luminescence spectra of various fluorophores, the solvatochromic phenomenon can be utilized in a wide range of applications such as biological essays. For example, enzyme-labeled fluorescence as a solvatochromic indicator can detect the proteins and nucleic acids in normal and cancer cells by binding to the cell sites as an external fluorescent [157]. In general, the spectral properties of most solvatochromic compounds are based on a donor-acceptor mechanism.

There are two types of solvatochromic systems based on this mechanism, one of which is a donor-bridge-acceptor (DBA) system, and the other is a donor-acceptor (D-A) system [158]. 

Solvatochromism is currently being widely investigated for optical probes and sensor applications such as solvatofluorochromism, liquid analysis, biological essays, polymers, and polymer-bound probes [159,160]. In the case of a liquid system, solvatochromism can be used to detect small concentrations of polar molecules in a non-polar system, e.g., methanol in naphtha. Solvatochromism can also be used to investigate other solvent parameters for assessing solubility phenomena and predicting suitable solvents for specific applications.

Solvatochromic materials can be applied to a polymeric system to investigate polar properties at the molecular level through polymers and polymer-bound probes. Accordingly, two different approaches are possible:

- embedding of the chromophore in the polymer chain.
- using a solvent containing a chromophore incorporated into the polymer matrix [137].

Solvatochromism can be used to detect explosives using carbon nanotubes as an emerging procedure through which, upon exposure to explosive particles, the frequency of emitted light from the nanotubes’ changes.

Furthermore, solvatochromic materials, used to develop a humidity-responsive system called a hydrochromic material, are also employed in functional inks and textile colorants for industrial applications [161].

### 3.8. Biochromic Materials

A biochromic compound is a type of chromogenic material that changes color through biochemical or hydrolysis reactions upon exposure to a biological stimulus. This mechanism is used to prepare a chromogenic system for the optical detection of microbial enzymes and reactions. As biochromic materials are not new, such materials are widely used for detecting pathogenic microbes. Numerous studies can be found on this topic and it has been reported that modified conjugated polymers, such as polydiacetylenes (PDA) and polythiophenes, can be used as biosensors in the form of liposomes (vesicles), thin films on solid supports, and membranes [162,163].

Conjugated polymers are suitable for different platforms embedded in various biological systems. The first report on conjugated polymers for a biological application was reported by Charych (1993) and was based on colorimetric detection using a biochromic system. A ligand-functionalized conjugated polymer experienced a colorimetric transition in a polymer matrix from colorless to red after interaction with a bio-stimulus [164].

**Figure 9:**
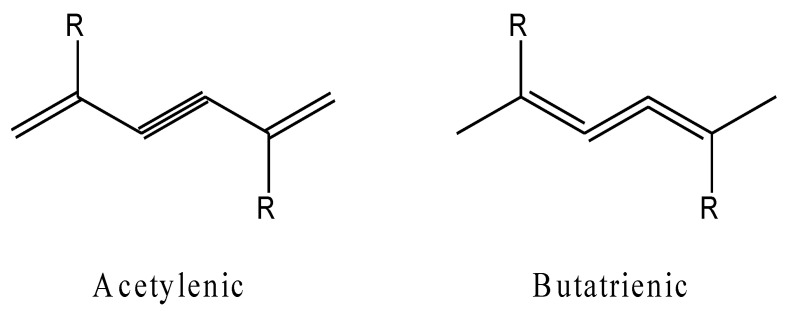
Resonance structures of polydiacetylene (PDA) backbone.

Natural lipids embedded in the polydiacetylene (PDA) matrix provide the platform showing the chromic transition due to alternating structural modifications of double and triple bonds in conjugated PDA polymer backbones following biochromism (Figure 9).

The mechanism in this system is that lipids function as recognition components that interact with environmental factors, such as biomolecules, and subsequently change the chain arrangement of double and triple bonds, as well as the electrical properties of the polymer matrix from acetylene to butatriene, leading to an end-to-end transition from blue to red in the liposomes [165].

PDA-based systems respond not only to environmental factors such as ligand-receptor interactions, temperatures, pH, mechanical stresses, and solvents but also to biological factors such as viruses, antibodies, microbial toxins, and bacteria [166].

Moreover, the detection of interfacial processes using membrane-mimics, including the detection of physiological ions, antibody-antigen binding, peptide–membrane interactions, and enzymatic catalysis, are potential applications of a PDA chromogenic system [137].

This dye class can be exploited for various potential applications including in rapid test kits for diseases, such as influenza, the detection of foodborne agents, such as *Escherichia coli* (*E. coli*) and other pollutants, the monitoring of DNA hybridization, and in drug research and development [167,168].

## 4. Smart Material Applications in Healthcare Technology

Table 4 summarizes the different categories of chromogenic sensing systems and smart materials described in the previous paragraphs, thus emphasizing the characteristics and recent applications in healthcare of chromogenic wearable sensors.

In light of the versatility and sensitivity of these systems for sensor devices, in the next paragraphs, thorough attention is devoted to the employment of halochromic materials in wearable and healthcare applications.

### 4.1. Applications of Halochromic Dyestuffs in Health Monitoring Sensors

pH sensors assume certain importance due to their ability to provide information about different processes and their applications in different fields, thus allowing their use in many areas such as biomedical, biotechnological, environmental, or food monitoring [190].

Recently, increasing awareness of health and wellness, together with the desire for higher athletic performance, has pushed scientists and entrepreneurs to design and develop devices for the continuous monitoring of health and physiological parameters for the early detection of pathologies, as well as the full control of factors affecting sporting achievements. Furthermore, since the medical management of non-critical pathologies is shifting toward the home-based monitoring of the patient condition rather than the current hospital-focused model, an exponential increase in user-friendly devices for the detection and measurement of bodily parameters is expected over the next few years. Among physiological signals, such as heart rate, blood pressure, and body and skin temperature, real-time sweat analysis has potentially opened a route for gathering valuable information about the physical and biological conditions of the individual [191]. Naturally generated by the human body, sweat is a transparent biofluid showing a pH of around 6.3, which is slightly more acidic than blood [192], representing an easily analyzable and accessible body fluid. Thanks to the various biomarkers contained in sweat, changes in pH could be used to identify several health disorders including acne, contact dermatitis, diabetes, and cystic fibrosis, among others [193]. A rise in sweat pH is observed in response to an increase in the sweat rate [194] or during metabolic alkalosis [195] in isolated sweat glands, and since a relationship between the sweat sodium ion concentration and pH has been found, this confirms that the greater the sweat [Na^+^] concentration, the higher the sweat pH. Moreover, in monitoring the pH of athletes’ sweat under dehydrated conditions, increased levels of Na^+^ have been observed [196], allowing athletes to promptly act to re-mineralize their bodies during exercise and sports competitions to enhance athletic performance [197].

Intelligent textiles for pH detection can be useful for the determination of alkaline or acidic conditions for monitoring the degree of environmental pollution or for medical tests [198]. The analysis of sweat pH is of paramount importance in fitness and athletic training due to the possibility of real-time monitoring sweat pH during physical activity, allowing the definition of specific approaches for rehydration and re-mineralization, which is crucial for endurance sport performance. In the medical field, the variation in sweat pH plays a role in the pathogenesis of skin diseases such as atopic dermatitis, irritant contact dermatitis, acne vulgaris, ichthyosis, and Candida albicans infections [199]. Furthermore, changes in the sweat pH create a favorable environment for the growth of some bacteria [200].

The first pH sensors were based on potentiometric and amperometric measurements and were often characterized by instability and drift, and sometimes needed constant recalibration [201]. On the contrary, the development of a fabric containing an indicator dye, also defined as a halochromic dye, capable of changing color in the visible radiation range, can be easily associated with an optoelectronic circuit and thus act as a pH sensor.

An optical sensor is schematically characterized by a sensing element (e.g., the indicator dye) and a transducer. A proper indicator, embedded in a specific configuration into a polymer matrix, is able to indirectly measure the concentration or presence of an analyte by producing an optical (e.g., colorimetric) variation, which is further converted into a measurable signal by the transducer element [202]. Different optical properties can be considered for optical sensors such as absorbance, reflectance, fluorescence, and luminescence, covering different regions’ spectra.

On the one hand, optical pH sensors show several advantages with respect to electrochemical pH electrodes, such as a longer lifetime, safety, low costs, fast and reversible responses, mechanical robustness, and ease of miniaturization [203]. In particular, they do not suffer from electromagnetic interference and are characterized by a higher signal-to-noise ratio (SNR). On the other hand, optical pH sensors present some disadvantages due to interferences or the intrinsic nature of the components. In fact, some sweat components, as well as ambient light, can influence the selectivity of the sensor, and leaching or photobleaching of the indicator can limit the sensor’s long-term stability. Moreover, the response time is a function of the mass transfer of the analyte from the sample to the indicator [204].

The immobilization of an indicator dyestuff onto fabrics ensures the possibility of developing a robust, reversible optical chemical sensor. In general, the polymeric matrix employed for the realization of a pH sensor should assist the proton diffusion (it should be hydrophilic) and is characterized by thermal, mechanical, and chemical stability. Furthermore, an efficient pH-sensitive dye should feature a high photostability and extinction coefficient, as well as a proper pKa [205].

Different methods can be conceived for the indicator immobilization into a polymer matrix, thus affecting the sensing behavior of the functional material itself. In this regard, the sol–gel technique represents a simple and efficient way to immobilize a halochromic dye onto textiles. In contrast to other methods with higher environmental impacts, the sol–gel technique is a sustainable alternative to conventional wet finishing processes [206].

In general, the use of the sol–gel technology for the realization of pH sensors may introduce some limiting factors, such as slow molecular diffusion into the dried silica-xerogel that leads to longer response times or reduced dye sensitivity due to covalent dye immobilization. In fact, the sensing behavior of the immobilized pH-sensitive dye in the polymer matrix can be perturbed because of the poor interaction with the textile sample.

### 4.2. Chemical Functionalization of Azo Dyestuffs for Immobilization on Textiles

The sol–gel technique has been used to produce textile surface modification to realize technical textiles, thus improving the ordinary performances of the treated fabrics. Various functionalities, such as UV-radiation protection, dye fastness, anti-microbial finishing, water repellency, bio-molecule immobilization, flame retardancy, and self-cleaning properties [207,208,209,210,211,212], have been achieved by depositing sol–gel coatings on a textile substrate. Furthermore, sol–gel materials have also been studied for innovative applications regarding, for example, hydrogen production by water photo-splitting and optical sensors [213]. This eco-friendly technique has several advantages over other methods of film deposition and allows the development of a thin protective hybrid layer on textile surfaces with well-defined physical characteristics, excellent chemical stability, and optical transparency, enabling the preservation of the mechanical and chemical properties of the fibers and introducing durable and high-performance functionalities [214]. Furthermore, the sol–gel film does not have cytotoxic effects on human skin cells, which is necessary for the employment of this technology in biological and medical applications [215].

The sol–gel method is based on the hydrolysis and condensation reactions of metal alkoxide precursors. Most of the literature refers to the sol–gel reactions for preparing silica coatings, even though this technology can also involve other inorganic precursors such as Al, Ti, Zr, Sn, V salts, or other alkoxides and organometallic substances in which the metal is linked to organic moieties that can be hydrolyzed and then condensed [216]. Nevertheless, Si precursors are most commonly involved in the realization of hybrid organic–inorganic materials despite their typical lower reactivity with respect to other alkoxide precursors. The sol–gel process is based on a phase transition of a liquid system (sol) into a dense one (gel), which is converted to a hybrid organic–inorganic porous matrix after further drying and heating [217].

**Figure 10:**
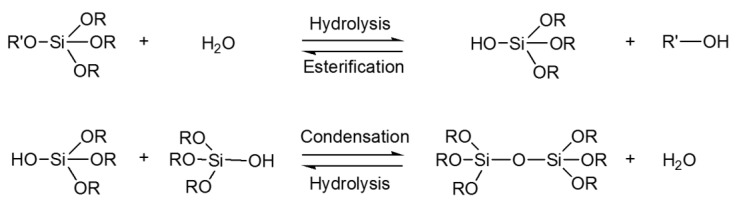
Schematic representation of hydrolysis and condensation of a silane alkoxide precursor [123].

The interaction between organic and inorganic precursors takes place thanks to weak bonds, such as ionic, hydrogen, or van der Waals, as well as covalent, coordination, or ion-covalent bonds [218,219]. In particular, the covalent interaction between organic and inorganic moieties leads to the obtainment of supramolecular hybrid organic–inorganic materials (HOIM). In this regard, organofunctional alkoxysilane precursors have aroused much interest for the chemical modification of fabrics and as useful crosslinkers. Functional silanes are characterized by a general chemical structure R′n-Si(OR)_4-n_, where the R′ groups are the alkyl or organo-functional groups, and the R fragments are mainly the methyl, propyl, or butyl groups. In particular, the hydrolyzable and polymerizable R groups are involved in the realization of the well-oriented 3D network structure together with the extra functional groups, such as the epoxy, vinyl, or methacryloxy groups [220,221,222], which are in charge of grafting with the fibrous substrate [223]. As shown in Figure 10, silane alkoxide precursors undergo hydrolysis in water, catalyzed either by acids or bases, and then parallel condensation reactions take place, leading to the network formation bearing the Si–O–Si bonds.

**Figure 11:**
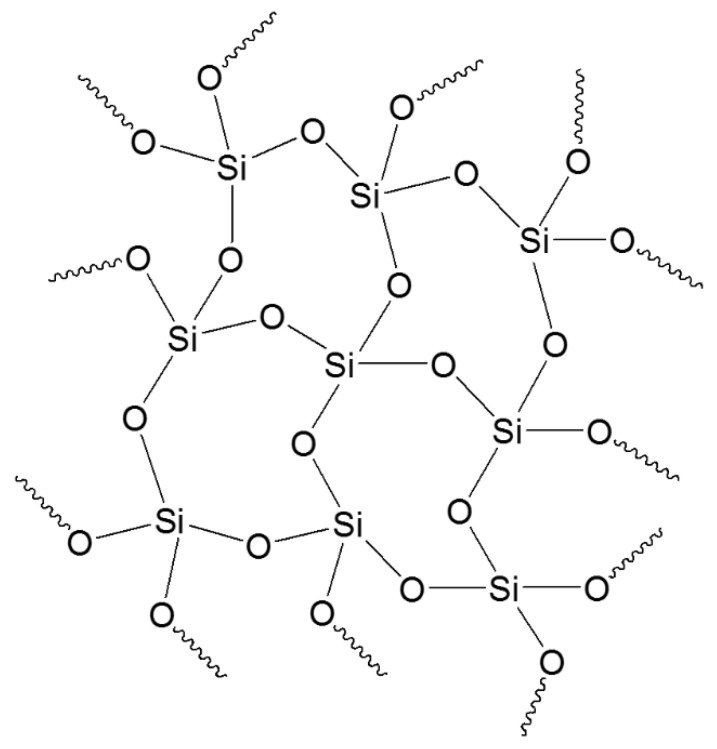
Three-dimensional network formed by subsequent condensation reactions.

The sol–gel technique shows many advantages, such as a mild process temperature, high purity of the obtained products, and easily removable solvent during the handling period. By tailoring the synthesis conditions, such as the concentration, temperature, time, pH, reactant ratio, solvent, and catalyst, the physical properties of the final inorganic matrix can be controlled to obtain different porous materials such as glass, polycrystalline powder, dry gel, or coating films [224]. The type of catalyst affects both the sols’ pH and the nature of the sol–gel polymer aggregates, allowing the realization of films, powders, or monoliths [225,226,227,228] (Figure 11).

Thanks to the low temperature of the sol–gel process and the porosity of the formed 3D network, the inorganic matrix may be doped with different organic/inorganic functional molecules, producing hybrid materials with peculiar functional performance. In particular, in the case of an anchored halochromic dye, it is still able to detect chemical changes in the environment revealed through color-developing reactions between the immobilized dye and an external diffusible chemical species. For this reason, smart textiles have attracted much interest as colorimetric sensors that are able to change color according to specific external stimuli. In particular, in the last years, many research groups have investigated the possibility of immobilizing indicator dyes using the sol–gel technique to realize optical sensing, for instance, for pH, ammonia, ionic species, oxygen, and VOC [229,230]. The dye reagent is easily incorporated into the porous silica-based network according to weak or covalent interactions. Weak interactions include hydrogen bonds or polar interactions and represent a simple and very versatile strategy, with the limitation of being weakened by the presence of water. On the other hand, the covalent bond can lead to the modification of the organic molecule but forms more stable interfaces compared to chemical doping. Among the numerous incorporable molecules into the sol–gel matrix, biological or enzymatic functional molecules, halochromic dyes, and liquid crystals are mostly used in optical and electro-optical applications [231]. The obtained color sensor can also be coupled with a read-out system [232].

Furthermore, in recent years, glycidyl methacrylate (GMA) has been extensively investigated for the functionalization of many polymers such as chitosan, cellulose, and starch [233,234,235,236,237]. The GMA monomer shows dual functionality resulting from its epoxy and acrylate moieties. The epoxy group can react with many other groups, such as amino, hydroxyl, and carboxyl groups, leaving the acrylate group available to graft onto cellulose through radical reactions [238]. This strategy has allowed the introduction of different functionalities into cotton fibers, conferring high durability to the treatment. Recently, UV-curing of glycidyl methacrylate monomers was proposed to promote in situ grafting of a methacrylic polymer network onto the surface of natural cellulose fibers to develop both composite polymer membranes [237] and wearable pH-meter textiles [239].

Recently, a simple strategy for preparing new stimuli-responsive cellulosic fabrics through the chemical modification of cellulose has been reported [240]. Although the grafting of acrylate monomers onto cellulose has already been extensively reported under heterogeneous conditions, the thermal-induced grafting of glycidyl methacrylate as a crosslinker of a pH-sensing dyestuff onto cellulose has been reported for the first time in the literature. The halochromic 1-((2,4-dinitrophenyl)diazenyl)-4-hydroxynaphthalene-2,7-disulfonic acid disodium salt (Nitrazine Yellow (NY)) was selected as an azo pH indicator and was able to change color from yellow to blue with a pH range between 6.0 and 7.0. Furthermore, compared to other halochromic molecules, NY dyestuff also features unique thermochromic properties, changing color from blue at 25 °C to green above 33 °C [241]. Such behavior could lead to the development of wearable multifunctional sensors able to detect pH and temperature changes. To promote a durable linkage between the dyestuff and the textile surface, minimizing the leaching of the dye, NY was functionalized by applying a coupling agent able to act as a linker through stable covalent bonds either with the cellulose matrix or the pH-indicator molecule. Without dye functionalization, plain NY dyeing (i.e., by physical adsorption) causes prominent dyestuff leaching from the cotton fabric after washing; as a matter of fact, despite the presence of a sulphonyl group in the molecular structure, pure NY is not able to bond covalently to the cellulose polymer chain.

### 4.3. Recent Examples of pH Sensor Development Based on GPTMS Dye

**Figure 12:**
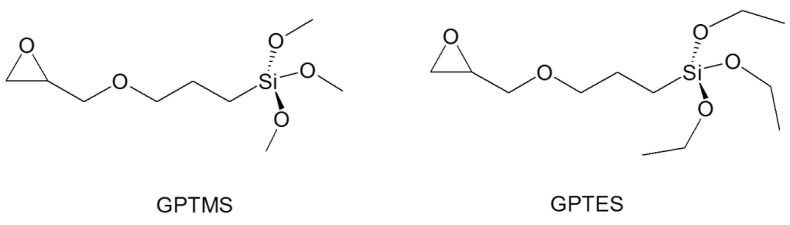
GPTMS (on the **left**) and GPTES (on the **right**) chemical structures.

The choice of the silane precursor is of crucial importance in sol–gel technology because it defines the features and the morphology of the resulting 3D matrix. The epoxy group is one of the most common organic ends in Si precursors, such as (3-glycidoxypropyl)trimethoxysilane (GPTMS) or its ethoxy derivative (GPTES). As shown in Figure 12, these sol–gel precursors combine the ability to crosslink organic molecule binders (through the reactive epoxy functionality) and the textile substrate (through the hydrolyzed Si–OH groups) [242,243]. Among the wide range of silane precursors, GPTMS has aroused much interest for its dual functionality (epoxy and methoxy silane). It can be considered a useful starting point for the design and realization of many functional hybrid textile materials by the embedding of dyes or functional organic/inorganic molecules in the sol–gel matrix. In fact, through catalyzed epoxy ring opening, organic molecules can be immobilized, whereas the condensation of the hydroxyl groups, deriving from the hydrolysis of methoxy-silane functionalities, allows GPTMS to act as a crosslinker toward cellulose, creating a 3D polymeric network on the fiber surface.

**Figure 13:**
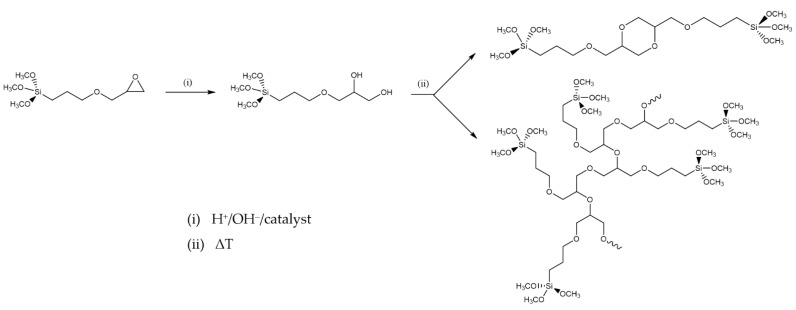
GPTMS epoxy ring opening through diol and formation of dioxane and/or polyether [245].

After the epoxy ring-opening and hydrolysis reactions, the epoxy group of GPTMS can result in the formation of diols, which can then result in poly-hydroxy ethers or polyethylene oxide 3D networks (Figure 13), in which organic molecules can be firmly immobilized in the sol or the cured xerogel grown on the solid surface [244,245]. 

Due to the immobilization of chemical sensing substances in the sol–gel matrix, this study intends to demonstrate the potential of GPTMS for the development of wearable sensors. In this area, several studies have examined the application of various halochromic dyes in sol–gel technology (see Table 5) [206,242,245]. 

The most common substrate for the conception of wearable textile-based sensors is cotton due to its favorable properties, such as biocompatibility, great mechanical strength, hydrophilicity, and so on, which makes cotton the ideal choice for many bio-applications [246].

However, synthetic fibers can be a suitable substrate for sol–gel functionalization. As reported by Van der Schueren et al. [247], sol–gel technology has shown to be efficient in developing polyamide-based (PA) halochromic sensors. Furthermore, GPTMS and Methyl Red (MR) were reacted in an ethanol solution, with a designed synthetic strategy aiming at forming a covalent bond between the silane precursor and the dye.

**Table 5:** Overview of scientific studies based on the immobilization of a halochromic dye into a GPTMS network for applications in the field of wearable pH sensors.

Catalyst	Dyestuff	Risk	Polyamide	Cotton
HCl	Methyl Red 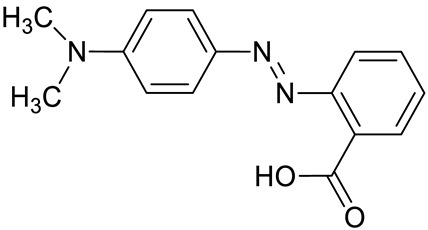	Azo dye used as pH indicator and for Methyl Red test. According to the International Agency for Research on Cancer (IARC), it is classified in group 3. Moreover, it is toxic for Gambusia affinis (LC 50: 7 mg/L–96 h). The breakage of the azo bond due to biodegradation can result in the release of toxic intermetabolites [248].	[247]	[232] [247] [243]
BF_3_OEt_3_	Nitrazine Yellow 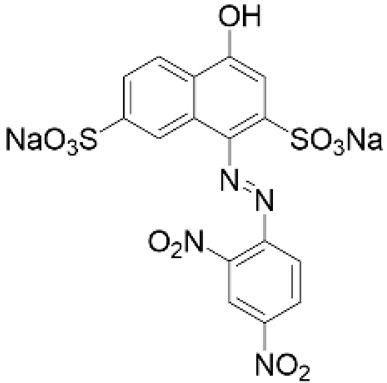	pH-sensitive azo dye largely employed in cell biology, histological, and hematological stains. It is harmful and can cause life-threatening diseases and skin irritation [128].		[240]

In a second step, after the addition of water, the hybrid network was formed through hydrolysis and condensation reactions. The sol–gel solution was applied to polyamide (PA) and cotton (CO), and the sol–gel technique and conventional dyeing were compared in terms of washing fastness. Each treated sample was washed according to the norm ISO 105-A01:2010 to evaluate the color fading. The color differences were measured by the grey scale and were in a range of 5 (best performing, low color fading) to 1 (worst performing, high color fading, Table 6).

**Figure 14:**
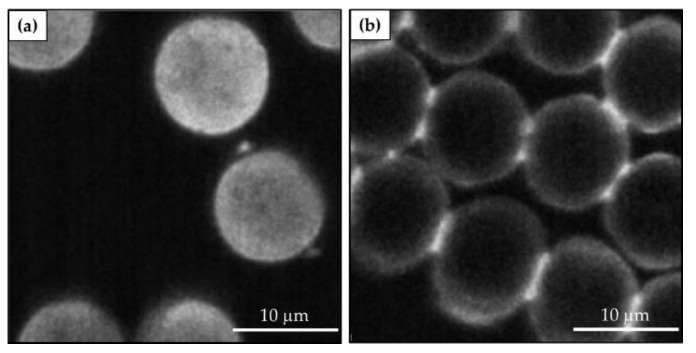
Confocal Laser Scanning Microscopy (CLSM) images relative to a cross-section of PA fibers showing the distribution of Methyl Red for conventional dyeing (**a**) and sol–gel treatment (**b**) [247].

Compared to traditionally colored PA, PA treated using the sol–gel technique demonstrated improved washing fastness and a more prominent halochromic sensing ability. Particularly, the former had a shorter reaction time because the sensing molecules were localized exclusively on the fiber surface, whereas conventionally colored PA demonstrated a slower response time because the dye was absorbed into each individual fiber (Figure 14). 

Color fading, after washing cycles, affects the change in the reflectance intensity of the halochromic dye and impairs the effectiveness of the pH sensor. However, this effect was very limited in the case of sol–gel treated fabrics thanks to the high washing fastness, which suggested insignificant further dye leaching in subsequent washing cycles. Moreover, the textile substrate did not significantly influence the halochromic response of the sol–gel coated samples, enabling the application of this technology to various textile fibers [247].

**Figure 15:**
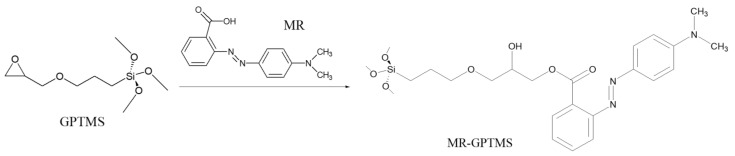
Schematic representation of the formation of Methyl Red–GPTMS through a covalent bond [232].

As already mentioned, the real-time measurement of the protonation–deprotonation process can be exploited to develop integration between coated fabrics and electronic devices. For example, a synthetic sol–gel strategy was employed by Caldara et al. [232] to realize an optoelectronic colorimetric pH sensor by taking advantage of the covalent bond established between Methyl Red and the epoxy ring of GPTMS (Figure 15).

**Figure 16:**
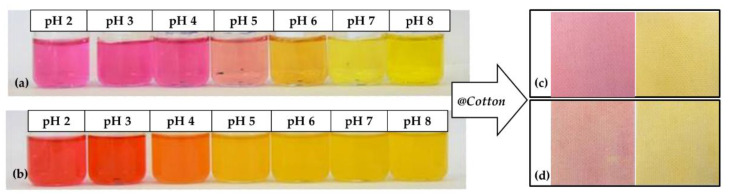
Methyl Red dissolved in buffer solutions (**a**), pH scale of Methyl Red–GPTMS sol (**b**), cotton fabrics coated with Methyl Red (**c**), and cotton fabrics coated with Methyl Red–GPTMS sol (**d**) [249].

Furthermore, besides reducing the leaching of the dyestuff from the silane matrix, the covalent bond in Methyl Red–GPTMS still allows the diffusion of H^+^ ions. The dyestuff color change is based on protonation in a range of 4.4–6.0, varying from red in an acidic solution to yellow in an alkaline or neutral solution, depending on the changes in its molecular and electronic structures (Figure 16). The covalent immobilization of Methyl Red does not alter the pH performance of the sensor (Figure 16b) compared with pure Methyl Red (Figure 16a). The same behavior is shown by cotton fabrics treated with the Methyl Red–GPTMS sol (Figure 16d) and with plain Methyl Red (Figure 16c).

**Figure 17:**
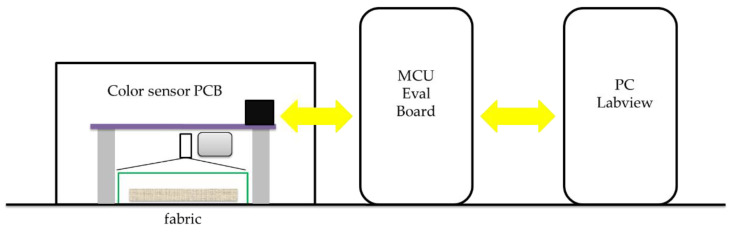
Fabric characterization system setup.

The color-sensing device, which is based on a white LED and a photodiode with monotonic spectral sensitivity, was developed to implement a light-to-frequency conversion. As a result, the color-sensing electronic system can measure the change in the fabric reflectance intensity. At the same time, it is wearable, has low power consumption, and is easily connectable to a microcontroller for data processing. The characterization system setup is shown in Figure 17.

All experimental findings confirm the use of the halochromic system as a promising wearable pH sensor for sweat monitoring. In fact, the pH response of the fabric was shown to be in a range between 3 and 8, showing a color settling time of the H^+^ variation of a few seconds when wet and a few minutes when dry, with a monotonic relationship between the color sensor frequency output and the pH. For pH 4.0–6.0, the system resolution is better than pH 0.05, with color reproducibility within 2% [232].

In another experiment, Caldara et al. [250] developed a wireless and non-invasive wearable sensor platform for sweat pH and skin-temperature monitoring. The wearable miniaturizing platform is based on a smart halochromic textile using Litmus as a non-toxic dyestuff in contact with the skin and a novel silicon-based thermometer. The platform was based on a smart halochromic textile sensor and has an electronic system that includes a thermometer, an RGB color sensor, a low-power microcontroller, a non-volatile memory, a Bluetooth miniaturized module, and a power supply. Furthermore, the designed wearable pH halochromic and temperature sensors were designed to achieve high resolution and accuracy while keeping power consumption low, with pH measurement accuracy better than ±0.4 pH and temperature resolution better than 0.01 °C, respectively. These interesting results demonstrated the potential of the developed wearable platform in monitoring athletes’ conditions to evaluate the dehydration grade or other important analytes [251].

In this regard, the same research group optimized and validated the non-invasive wearable pH sensor by conducting on-body experiments [251].

**Figure 18:**
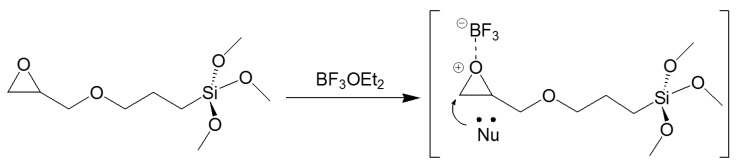
Schematic of BF_3_OEt_2_ as Lewis acid, catalyzed mechanism of epoxy ring opening [242].

The synthetic strategy was based on the interaction between GPTMS and the dyestuff. The work was focused on selecting specific catalysts for epoxy ring-opening polymerization, which can be achieved by cationic, anion, or covalent nucleophilic mechanisms. Among the catalysts, the role of BF_3_OEt_2_, which can act both on epoxy ring opening and on Si–O–Si network formation depending on its concentration, has been investigated. The epoxy ring-opening mechanism assisted by BF_3_OEt_2_ catalysis is shown in Figure 18.

**Figure 19:**
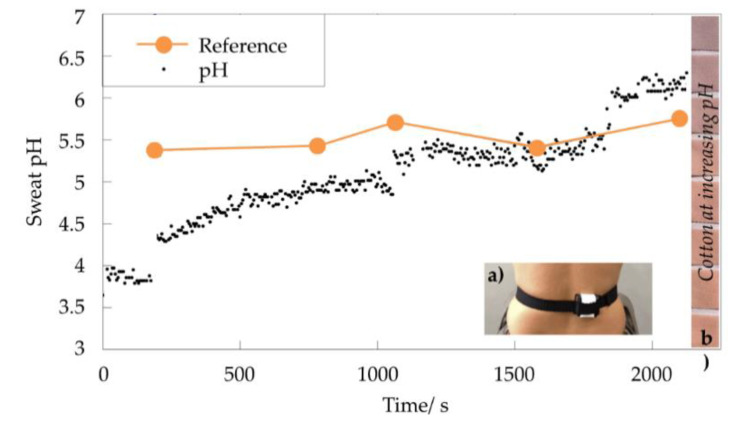
Comparison between five local skin pH (reference pH) and on-body continuous sweat pH measurements during physical activity [251].

In particular, Litmus was entrapped in the GPTMS-based 3D network using BF_3_OEt_2_ as a catalyst for epoxy ring opening (GPTMS: LITMUS molar ratio 10:1). The stable interaction between the sol–gel-based matrix and the dyestuff avoided interference in the colorimetric detection of pH due to dye leaching and ensured the proper features for sensor applications such as mechanical stability, porosity, thickness, and uniformity. To obtain a pH estimation model, the resulting halochromic hybrid sol–gel was tested in a buffered solution in the sweat pH range. A miniaturized electronic device (Figure 19), featured by a color sensor with high sensitivity, low power, embedded processing, and a Bluetooth interface, was combined with the coated textile [251].

The sensor was tested on-body during bike exercises by measuring the sweat pH five times and simultaneously with a reference electrochemical pH meter. The results obtained by the wearable platform showed a resolution of 0.2 pH and 0.5 pH, in agreement with the reference pH meter, with a settling time of 8 min because of a poor sweat rate at the beginning of perspiration [251].

**Figure 20:**
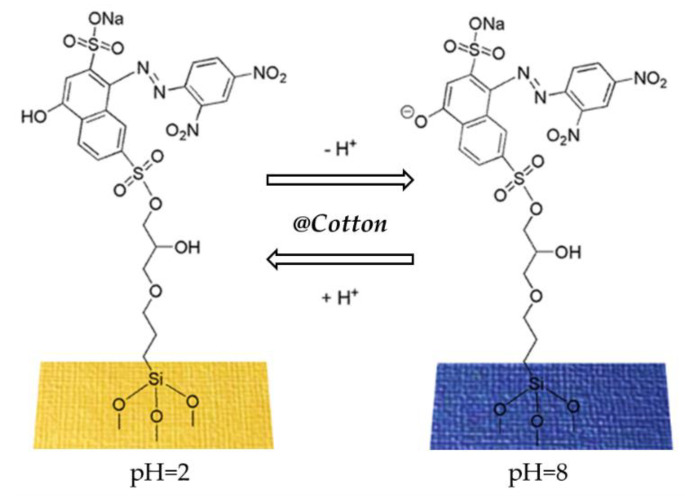
Halochromic pH-sensitive properties of Nitrazine Yellow–GPTMS immobilized onto cotton fabric [242].

Guido et al. [242] confirmed the influence of the BF_3_OEt_2_ as a catalyst for developing sol–gel-based halochromic pH sensors. Three different concentrations of Lewis acid were used (1%, 5%, and 10% *w*/*w* GPTMS) for the sol–gel synthesis, and the same reactions were conducted by adding Nitrazine Yellow (NY) to immobilize it onto cellulose (GPTMS:NY molar ratio 20:1). The halochromic hybrid GPTMS solutions were padded onto cotton fabrics and the experimental findings demonstrated that the pH-sensitive properties of Nitrazine Yellow were maintained even after covalent immobilization in the silane matrix through its sulfonic group (Figure 20). The high concentration of BF_3_OEt_2_ provided the best results for the formation of the inorganic network crosslinking structures that enhanced the fixation of the hybrid matrix onto cotton. These results also demonstrated a stronger halochromic response and washing fastness of the realized textile sensor [242].

**Figure 21:**
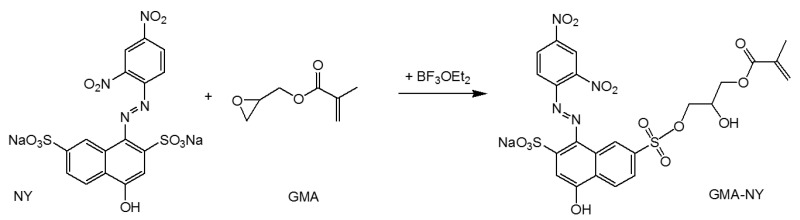
Schematic of the synthetic pathway for the formation of the functionalized GMA–NY dyestuff.

Moreover, a bifunctional precursor, glycidyl methacrylate (GMA), containing epoxy ring functionality and an acrylic group, was employed to anchor NY on the open-ring GMA moiety in a catalyzed BF_3_OEt_2_ reaction step (Figure 21). According to previous research focused on the covalent sol–gel immobilization of NY through the ring opening of epoxy alkoxysilanes [242], BF_3_OEt_2_ was used as a catalyst at the highest investigated concentration (10% *w*/*w* GMA) since the pH indicator reaction with BF_3_OEt_2_ reduces the availability of the catalyst in aqueous solution [242]

The NY covalent linkage with the GMA bifunctional monomer was performed through a BF_3_OEt_2_-catalyzed epoxide ring opening of the acrylate monomer, thus obtaining the modified GMA–NY derivative. The latter was fully characterized by NMR, ATR–FTIR spectroscopy, and UV-Vis analysis to investigate its chemical structure and halochromic properties. The NMR investigation confirmed the real para-hydroxyazo structure of the NY dyestuff and the preferential nucleophilic site, namely at C6 carbon for the substitution reaction by the GMA epoxy functionality. The effectiveness of the catalyzed epoxy ring opening of the bifunctional monomer was further confirmed by the ATR–FTIR analysis of GMA–NY. According to UV-Vis spectroscopy, the pH response of the functionalized NY was consistent with the typical halochromic response of the plain NY with a pH in a range of 4–8. Potassium persulfate was employed as the radical initiator in the grafting of the GMA–NY onto the cellulose substrate through the exhaustion method. The grafted fabrics were thoroughly investigated by ATR–FTIR to assess the durable adhesion of the coating after one and five washing cycles, thus confirming both the efficacy of the grafting immobilization onto cotton and the good washing fastness of the coating. UV-Vis diffuse reflectance spectroscopy and CIELAB measurements revealed that the typical halochromic behavior of the NY dyestuff was maintained on grafting onto cotton, although the covalent immobilization determined a bathochromic maxima shift compared to the corresponding spectra recorded in the solutions. The CIELAB measurements demonstrated the reversibility of the halochromic properties of the immobilized NY onto cotton and their repeatability after different exposure cycles, thus evidencing the non-disposable nature of the designed wearable pH sensor. The dyestuff grafting onto cotton using a thermal radical initiator proved to be an efficient strategy to obtain stable and robust halochromic wearable sensors, thus representing a useful, long-lasting, and cheap alternative to conventional dyeing techniques.

**Figure 22:**
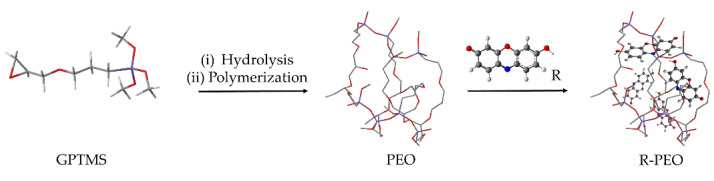
Schematic representation of the formation of PEO followed by the immobilization of the Resorufin (R) through hydrogen bonds or weak electrostatic interactions (R-PEO) [245].

A cellulose substrate was also photografted with Nitrazine Yellow after it had been functionalized with GMA in the presence of a photoinitiator [239]. An ecologically friendly substitute for the BF_3_OEt_2_ catalyst was the photo-induced method. Using CIELAB color changes and UV-Vis reflectance measurements, the halochromic and pH responses of treated cotton were examined. The cloth changed color when exposed to acidic and alkaline environments (both in wet and vapor conditions), which could be seen by the naked eye and was clearly recognized by analytical tools. It was thus shown that the modified GMA–NY dye, photografted on cotton surfaces, can successfully impart pH-sensing characteristics onto the fabric, opening the door for numerous applications of the suggested approach in the field of smart textiles. To guarantee efficiency and durability, the immobilization of a halochromic dye onto a sol–gel matrix requires certain stability to avoid leaching phenomena and ensure a color response, similar to immobilization onto fabrics. Therefore, the covalent immobilization of a halochromic dye with GPTMS was an essential step for preventing leaching phenomena but it has been demonstrated that, in some cases, a non-covalent encapsulation of the sensing molecule into the 3D sol–gel network can be an alternative and new pathway. As demonstrated by Rosace et al. [245,252], the pH response of a Resorufin–GPTMS sol was evident even if the dyestuff was non-covalently immobilized into the polyethylene oxide (PEO) 3D network, formed by epoxy ring opening and the subsequent polymerization of the GPTMS (Figure 22). The halochromic sol–gel was synthetized by adding GPTMS to an ethanol solution of Resorufin (GPTMS:Resorufin molar ratio 30:1; 1-methylimidazole 5% *w*/*w* GPTMS) and the resulting solution was neutralized at pH 4 through a catalytic amount of HCl 0.1 M. The filtered solution was analyzed by UV-Vis spectroscopy to be in a pH range of 2–8, showing the typical halochromic behavior of immobilized Resorufin.

The halochromic solution was also applied to cotton fabrics resulting in a homogeneous coating with slight dye leaching in the washing tests, as shown in Table 7. Moreover, in agreement with the washing performance, no color leaching was observed, even after submitting treated textiles to pH changes (2 < pH < 8).

Photostability in visible light is a prerequisite for the discussed applications. Plutino et al. [243] conducted an interesting study in this area by analyzing the photostability of the complex Methyl Red–GPTMS sol under UV/Vis radiations with different pH values, observing a photostability increase under acid conditions. This behavior was associated with the electron density of the azo bond and the reduced extent of the hydrogen bonding between the azo groups in the complex (with respect to the unfunctionalized dye), which seemed to affect the reduction rate rather significantly. The photostability of the Methyl Red–GPTMS matrix was enhanced by the GPTMS binding to the ortho-phenyl position relative to the azo bond. This behavior allows using this class of dyes as halochromic sensors characterized by light fastness and efficiency [243,253].

In the literature, more applications of sol–gel technology for the encapsulation of pH indicators to realize halochromic hybrid textiles have been reported. As an example, Sun et al. [254] employed Methyl Red, Phenol Red, Bromocresol Green, and Bromothymol Blue in the synthesis of a hybrid matrix based on GPTMS (precursor: dyestuff molar ratio 1:200). The treated cotton samples were tested, and the experimental results demonstrated the importance of the size, affinity, and shape of the dyestuff for the interaction with the sol–gel network.

### 4.4. Wound (pH) Sensors

When wounds become chronic and do not heal properly, they represent a relevant financial burden on the patient and the healthcare system. The use of bandages to encourage the creation of a clean environment for quick healing processes is suggested because their restoration is a complicated process that is significantly influenced by numerous factors [255]. Monitoring the pH of a wound has been the focus of several recent studies [256] since pH shifts at the wound site can provide helpful information regarding wound evolution. Due to internal and external influences, a wound’s pH value varies over several phases, including healing, inflammation, and ulceration, due to the presence of various types of bacteria and enzymes. Although the pH of healthy skin is mildly acidic (pH = 4–6), it becomes more alkaline (pH = 9) when the skin barrier has been compromised.

A highly acidic pH could be a sign of a bacterial infection as the wound’s pH progressively reaches neutral during the healing process [257]. Therefore, monitoring the pH changes in wounds can provide information about the state of wound healing [258]. The development of methods for monitoring the activity of bacteria during the healing process, such as fluorescent dyes, anodized porous silicon, temperature, and pH, is currently driven by the fact that microbial proliferation is a substantial source of concern [42]. As reported by Cui et al. [259] many devices have already been investigated to continuously monitor wound changeable pH, such as electrochemical pH sensors, ion-sensitive field effect transistors, fluorescence spectroscopy, and so on. Complex wound settings, however, can have an impact on the signal output and decrease the sensitivity of this detecting apparatus. A wound dressing that changes color for different pH values can display an optical signal in a non-destructive manner and act as a pH sensor for visual monitoring. Halochromic dyes are typically utilized in this application. A halochromic dyestuff’s apparent color change is mainly caused by the changing electronic configurations that the dye molecules take on during protonation or deprotonation. Anthocyanins (4–12) and alizarin (5.8–13) are two other halochromic dyestuffs that have a larger pH response range and more noticeable color shifts between 5 and 9. Additionally, these are two kinds of halochromic natural dyes that have been shown to have some medical uses [260].

## 5. Final Remarks and Future Perspectives

Wearable chemical sensors have made significant advancements in recent years. Over the next ten years, the development of these devices is expected to expand quickly. Wearable chemical sensors have a promising future, but there are still numerous obstacles to overcome and technological gaps to close before they reach their full potential. By overcoming these obstacles, wearable chemical sensors that are low power and easily integrated with the body will become more commercially viable and continuously give the wearer helpful information in a user-friendly, safe, and secure manner.

Only innovative cross-disciplinary research by academics not only from traditional STEM disciplines but also from the Humanities can address some of the problems that researchers in the field of wearable chemical sensors are currently facing. Such interdisciplinary work will produce technologically sophisticated wearables that a wide range of consumers will be eager to employ in their daily lives. Scientists working on the related materials should, for instance, create new materials that will allow the conversion of traditional chemical sensors into wearable formats.

Similar to this, researchers working in the energy field should concentrate on creating wearable, biocompatible power sources with a high energy density and long life, and incorporating multisource energy-collecting devices. Engineers working on wireless communication networks should also create devices that will allow a high density of wearable electronics to communicate continuously at high data rates. Wearable sensors are expected to produce enormous volumes of personal data as the market grows, raising serious questions about data security and user privacy.

Therefore, next-generation algorithms must be developed by cryptologists to protect the data produced by wearable sensors. The smooth integration of these subsystems in addition to resolving the problems faced by the above-mentioned industries with regard to wearable chemical sensors must be addressed. Wearable chemical sensor systems have been impressively integrated as a result of recent investigations [64]. Given the particular challenges faced by the wearable sensor industry and the intricate relationships between the subsystems, such integration calls for brilliant systems engineering skills.

Different fabrication procedures for the various physical components of the entire system present integration challenges. Ineffective device integration is also caused by differences in subcomponent packing. For instance, although the supporting electronics must be totally sealed off from any exposure to moisture, the chemical sensor must be exposed to the biofluid. The interface between the sensor components is typically where the system as a whole is most prone to malfunction. Thus, the development of wearable chemical sensors depends critically on the seamless integration of these subsystems. The field of wearable sensors needs close coordination and collaboration among medical professionals in addition to the involvement of engineers and scientists. It is anticipated that wearable sensors will produce personal health data that was previously hard to access. In order to properly understand the data resulting from such health monitoring, close contact with physicians is necessary.

It is evident that the field of wearable chemical sensors offers exciting opportunities for collaboration, and the commercial success of this rapidly expanding field will ultimately depend on the ability of researchers to continue innovating and working together to address the problems that wearable chemical sensors currently face.

## 6. Conclusions

The development of wearable sensors is a research challenge with potential applications in many fields such as healthcare, fitness, medicine, security, military, and so on. Their potential for the real-time and continuous monitoring of patient physiology outside the hospital environment provides many useful advantages such as reduced pressure and costs for healthcare systems. The current pandemic has stressed the importance of developing telemedicine approaches for patients with chronic diseases requiring continuous control of their health conditions. However, wearable sensors embedded in garments must be robust and flexible as clothing is subjected to several mechanical deformations (bending, stretching, etc.); therefore, wearable sensors must maintain their sensing responses under these critical conditions.

This paper has reviewed stimuli-responsive materials and their applications as textile-based wearable sensors. In particular, the effective use and consequent benefits stemming from the immobilization of dye indicators in the sol–gel matrix for the design of wearable sensors have been discussed. The inclusion of sensitive molecules causing colorimetric variations depending on sweat pH is of great interest for the numerous applications that can be envisaged in various fields, such as medicine, fitness, wellness, and the environment. The combination of halochromic dyes and non-invasive electronic devices allows for their use for health monitoring, such as checking specific parameters during sports activities, and they have a low cost and easy usability. Research activities are continuously evolving and designing wearable sensors that can detect important analytes that may be of great importance in diagnostic and medical applications and, therefore, simplify current detection methodologies. It should be highlighted that developing smart textiles requires a multidisciplinary approach, in which chemistry and electronic knowledge are well integrated with a deep understanding of the modified functional textiles.

## Figures and Tables

**Table 1:** Types of electrochromic polymers.

Electrochromic Polymer	Types
Electrochromic polymers based on transition metal coordination complexes	Reductive electropolymerization of polypiridyl complexes Oxidative electropolymerization of polypiridyl complexes Metallophtalocyanine electrochromic films
Viologen polymeric systems	Polymeric viologen systems
Conjugated electrochromic polymers	Pyrroles and diocypyrroles Copolymers and n-dopable electrochromic polymers Functionalized electrochromic polymers and composites Polyanilines as electrochromic materials

**Table 2:** Functional groups, pH ranges, and color changes of the phthaleins, sulfophtaleins, and azo dyes reported in Figure 6.

Name	R^1^	R^2^	R^3^	X	pH Range	Color Change
Bromocresol Green	Br	Br	CH_3_	SO_2_	3.8–5.4	Yellow-blue
Bromocresol Purple	CH_3_	Br	H	SO_2_	5.2–6.8	Yellow-purple
Bromophenol Blue	Br	Br	H	SO_2_	3.0–4.6	Yellow-blue
Bromophenol Red	Br	H	H	SO_2_	5.2–6.8	Yellow-red
Bromothymol Blue	CH(CH_3_)_2_	Br	CH_3_	SO_2_	6.0–7.6	Yellow-blue
Chlorophenol Red	Cl	H	H	SO_2_	4.8–6.4	Yellow-red
Cresol Red	CH_3_	H	H	SO_2_	7.2–8.8	Yellow-red
Cresolphtalein	CH_3_	H	H	CO	8.2–9.8	Colorless-red
Phenol Red	H	H	H	SO_2_	6.8–8.4	Yellow-red
Phenolphtalein	H	H	H	CO	8.5–9.0	Colorless-red
Thymol Blue	CH(CH_3_)_2_	H	CH_3_	SO_2_	1.2–2.8 8.0–9.6	Red-yellow Yellow-blue
Thymolphtalein	CH(CH_3_)_2_	H	CH_3_	CO	9.3–10.5	Colorless-blue
Xylenol Blue	CH_3_	H	CH_3_	SO_2_	1.2–2.8 8.0–9.6	Red-yellow Yellow-blue
	**R^4^**	**R^5^**				
Methyl Orange	H	SO_3_H			3.1 4.4	Red Yellow
Methyl Red	CO_2_H	H			4.4 6.2	Red Yellow

**Table 3:** pH-sensitive dyes prepared from commercial products.

Types
Chromogenic anion sensors and metallochromism
Metallochromism in chelates and crown ethers
Fluorans
Leuco di- and tri-arymethanes
Phtalides
Azo and styryl dyes

**Table 4:** Smart materials together with the main characteristics and some recent examples of their application in healthcare technology.

Smart Material	Characteristics	Applications	Refs.
Chromogenic	Reversible, reusable, solid-state applications, stimuli-responsive, color-change detection to the naked eye	Eye patch biosensors for biomarkers in human tears, glucose monitoring, sweat monitoring	[169,170,171]
Photochromic	Color-changing capacity when exposed to light and sunlight (IR and UV radiations) can alter their optical characteristics; high temperatures can accelerate the material decomposition	Fluorimetric multi-sensing of sweat biomarkers, UV indicators, temperature, and sweat pH sensing	[172,173,174]
Thermochromic	Different color states at different temperatures, versatile	Human movement monitoring (strain), body temperature	[175,176,177]
Electrochromic	Sensitive to redox reactions, they have to feature a fast response to injection and ejection processes, coloration efficiency, high contrast level, specified life cycle, and write–erase efficiency	Skin temperature and wrist movement, alarm system for smart contact lenses, glucose sensing	[178,179,180]
Ionochromic	Color-changing ability by inducing ionic species in an ionic state, versatile, selective, different commercial applications	pH monitoring, acid gas sensing, ammonia gas detection	[181,182]
Mechanochromic	Optical-changing properties when subjected to mechanical stimuli, low pressure-responsive ability, versatile	Volatile organic compound (VOC) detection, subtle and large human motion sensing	[183,184]
Solvatochromic	Display different colors depending on the solvent in which they are dissolved, versatile, highly sensitive	Lactate sensing, sweat analysis	[185,186]
Biochromic	Changes color through biochemical or hydrolysis reactions upon exposure to a biological stimulus, can be exploited for various biological potential applications	Sweat sensing, glucose detection, in vitro perspiration monitoring	[187,188,189]

**Table 6:** Washing fastness of cotton (CO) and polyamide (PA) conventionally and sol–gel dyed. (1 = worst performing; 5 = best performing) [247].

Sample	Performance
CO conventionally dyed	1
CO sol–gel dyed	4
PA conventionally dyed	2/3
PA sol–gel dyed	5

**Table 7:** Add-on (%) after 1 and 5 washing cycles of cotton samples (CO) treated with Resorufin–GPTMS sol [245,252] together with their corresponding images.

Sample Code	Sample Image	Add-On (%)
CO_Resorufin-GPTMS	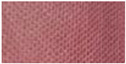	7.9
CO_Resorufin–GPTMS after 1 washing cycle	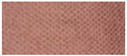	6.0
CO_Resorufin–GPTMS after 5 washing cycles	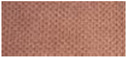	5.5

## Data Availability

Not applicable.
